# Passive Disease Surveillance of Alpine Chamois (*Rupicapra r. rupicapra*) in Slovenia between 2000 and 2020

**DOI:** 10.3390/ani12091119

**Published:** 2022-04-27

**Authors:** Gorazd Vengušt, Urška Kuhar, Klemen Jerina, Tanja Švara, Mitja Gombač, Petra Bandelj, Diana Žele Vengušt

**Affiliations:** 1 Institute of Pathology, Wild Animals, Fish and Bees, Veterinary Faculty, University of Ljubljana, Gerbičeva 60, 1000 Ljubljana, Slovenia; gorazd.vengust@vf.uni-lj.si (G.V.); tanja.svara@vf.uni-lj.si (T.Š.); mitja.gombac@vf.uni-lj.si (M.G.); 2 Institute of Microbiology and Parasitology, Veterinary Faculty, University of Ljubljana, Gerbičeva 60, 1000 Ljubljana, Slovenia; urska.kuhar@vf.uni-lj.si (U.K.); petra.bandelj@vf.uni-lj.si (P.B.); 3 Department of Forestry and Renewable Forest Resources, Biotechnical Faculty, Večna Pot 83, 1000 Ljubljana, Slovenia; klemen.jerina@bf.uni-lj.si

**Keywords:** disease monitoring, post-mortem examination, infectious diseases, non-infectious diseases, chamois

## Abstract

**Simple Summary:**

Wildlife disease surveillance can be considered an essential tool for providing important information about the health status of the population and for protecting human health. Between 2000 and 2020, 284 chamois carcasses from the entire home range of the species in Slovenia were examined using comprehensive necropsy and other laboratory tests. The results indicate a wide range of chamois diseases, but none of the identified diseases can be considered a significant health threat to other wildlife species and/or to humans.

**Abstract:**

In this paper, we provide an overview of the causes of death of Alpine chamois (*Rupicapra r. rupicapra*) diagnosed in the national passive health surveillance of chamois in Slovenia. From 2000 to 2020, 284 free-ranging chamois provided by hunters were necropsied at the Veterinary Faculty, University of Ljubljana, Slovenia. Depending on the results of complete necropsy, histopathological, bacteriological, parasitological, and virological examinations, a descriptive data analysis was performed. The most common causes of death in chamois were infectious diseases (82.2%), followed by non-infectious diseases (11.8%). Of all the causes of death, parasitic infections accounted for 70.3%, trauma for 9.7%, and bacterial infections for 9.3% of all cases. Less common diseases were viral infections, neoplasms, winter starvation, and metabolic disorders.

## 1. Introduction

The alpine chamois is a habitat-specialized ungulate that inhabits “continental archipelagos” with fragmented rocky habitats, often limited to high elevations [1]. In the late 1970s, the chamois populations in the Eastern Alps, including Slovenia, declined sharply due to catastrophic mange epidemics [2,3], with a local population decrease of up to 80%. Subsequently, the population began to recover, leading to a general increase in the density and distribution of the species in Europe [4], including Slovenia. The population size of the chamois in Slovenia has remained stable over the last two decades. Their number is estimated at over 10,000 individuals [5], and the annual culling rate averages 2900 individuals (X ± SD = 2286 ± 102) (unpublished data).

The occurrence of numerous infectious diseases in chamois has been documented in the scientific literature [6,7,8,9,10,11,12,13]. Frequent and close contacts between livestock and wild ruminants pose a risk of the cross-transmission of emerging and re-emerging pathogens and are a potential problem in the Alps due to traditional gazing in late spring and summer [4,7,14]. Therefore, the monitoring of circulating pathogens in wildlife populations is important in order to assess the causes and sources of disease and to understand its transmission between wild and domestic animals [8]. Palmer et al. [15] and Rossi et al. [4] have provided the evidence of pathogen transmission between wildlife and livestock. Bacterial infections, such as infectious keratoconjunctivitis [16,17]; viral infections, such as contagious ecthyma [9]; and parasitic infections, such as haemonchosis [10] or fasciolosis [18], have been found in wild ruminants that are likely transmitted from livestock. The One Health concept recognizes that human health is closely linked to animal and environmental health. Zoonotic diseases with a wildlife reservoir are usually caused by various bacteria, viruses, and parasites, while fungi are of secondary importance [19]. Studies indicate that, fortunately, none of the diseases found in chamois pose a significant health risk to humans or other wildlife species. To date, only sporadic cases of contagious ecthyma have been reported, which can be transmitted to humans following contact with infected animals or contaminated material [20]. Most studies in chamois are limited to selected infections. Some limited information known about host mortality has been studied in the context of general wildlife health surveillance, which is considered a valuable tool for early warning systems [21] and important information about the health status of wildlife populations [22].

In this study, we provide an overview of the passive disease surveillance of Alpine chamois in Slovenia between 2000 and 2020.

## 2. Materials and Methods

### 2.1. Samples

We analyzed the records of 284 necropsies of chamois carcasses (male, *n* = 182; female, *n* = 102) in Slovenia between 2000 and 2020, provided by volunteer hunters and professional game wardens from all over the country throughout the year (winter season *n* = 115; spring season *n* = 75; summer season *n* = 29; autumn season *n* = 65). Almost 59.5% (*n* = 169) of the carcasses were from chamois found dead in the wild, 35.5% (*n* = 101) of the carcasses were from diseased chamois (visible lesions, unusual behavior, paresis, weight loss, diarrhea, etc.) that were legally harvested, and 5% (*n* = 14) of the animals were harvested during the regular annual cull and subjected to necropsy due to suspected disease or other observed pathological lesions. The fresh or frozen carcasses were delivered to the Veterinary Faculty, University of Ljubljana, by the Veterinary Hygiene Services (VHS), which is responsible for the disposal of dead animals in Slovenia. The age of each animal was estimated from the number of horn annuli rings. We divided the animals into three age groups: kids and juveniles (under 1 year old), juveniles (1–2 years old), and adult animals (over 2 years old).

### 2.2. Laboratory Methods

Various laboratory methods were used to determine the causes of mortality or morbidity.

Protocols and procedures for animal necropsies, including those of wild animals, are available from a variety of sources. Complete necropsies were performed on all chamois carcasses. A detailed description of necropsy protocols and procedures in wildlife can be found in McAloose et al. [23]. The decision to collect specimens for additional laboratory testing depended on the history and results of the necropsy in each case. In general, samples were collected from selected parenchymal organs (lung, heart, liver, kidney, and spleen) and all pathologic lesions.

Tissue samples collected during the necropsy were fixed in 10% neutral buffered formalin, processed, embedded in paraffin, cut, and stained according to standard protocols with hematoxylin and eosin (H&E) and, for selected sections, also with the periodic acid–Schiff staining method (PAS), Gram and Ziehl-Neelsen. When necessary, additional special staining was performed for tissue-based diagnosis. For bacteriological examination, culture from the tissue samples was usually prepared on blood agar (5% sheep blood) and incubated aerobically and anaerobically at 37 °C. After 24 h of incubation, the blood agar plates were examined for the presence of pathogenic bacteria. If necessary, plates were incubated for an additional 48 h. Isolates were biochemically characterized using API (commercial system API bioMerieux, Marcy I’Etoile, France) and later MALDI-TOF MS (Matrix-assisted laser desorption ionization-time of flight mass spectrometry) (Bruker Daltronik GmbH, Bremen, Germany) according to the manufacturer’s instructions. MALDI-TOF MS was introduced into routine microbiological practice at the Veterinary Faculty in 2015.

Gastrointestinal tract, lungs, liver, abdominal cavity, and skin were examined for the presence of parasites. The stomach and intestines were removed, cut open lengthwise, rinsed with strong jets of water through 300-μm and 125-μm sieves, and examined macroscopically. The liver and lungs were cut into pieces and immersed in lukewarm physiological solution (0.9% NaCl solution). Parasites from the stomach (abomasum), intestine, and other organs were fixed in buffered formalin or 70% ethanol. Parasites were identified based on morphological and anatomical differences using microscopic image analysis as described by Soulsby [24], Niewiadomska [25], and Anderson et al. [26]. Species were identified based on the morphological characteristics of the reproductive organs of adult male nematodes, but body size, structure of the head end, tail end, oral capsule, were also used for morphological identification.

The diagnosis of sarcoptic mange was confirmed by skin scrapings and potassium hydroxide skin digestion and examined under a stereomicroscope. Detailed information on diagnostic procedures can be found in the study by Pérez et al. [27].

For detection of Orf viruses (ORFV) in skin lesions using PCR, skin tissue samples were stored at −70 °C until analysis. Ten percent suspensions were prepared from the tissue samples (1 cm^3^ of tissue was added to 9 mL of RPMI medium 1640 (Thermo Fisher Scientific, Carlsbad, CA, USA)). The suspensions were homogenized and centrifuged at 2000× *g* for 10 min. The supernatant was stored at −70 °C when not processed immediately. The supernatant was used for nucleic acid extraction using the DNeasy Blood & Tissue Kit according to the manufacturer’s instructions (Qiagen, Hilden, Germany). The PCR was performed as previously described by Kottaridi et al. [28] with a combination of primers ORF 045F (5′ cct act tct cgg agt tca gc 3′) and ORF 045R (5′ gca gca ctt ctc ctc gta g 3′). The PCR products were subjected to electrophoresis in a 1.8% agarose gel.

The cause of death or morbidity was determined based on the history, necropsy findings, and results of additional laboratory tests.

### 2.3. Evaluation of Representativeness of Surveillance

To assess the representativeness of passive sampling based on voluntary submission of samples by hunters, we analyzed (1) trends in the annual frequency of submitted samples over time, (2) their proportion and sex and age structure compared to the total recorded natural mortality of chamois, and (3) the spatial distribution of the submitted samples compared to the local density of chamois. For this purpose, we used data from the National Wildlife Mortality Register [5], which is compulsorily maintained by all managers of hunting grounds according to national legislation. The registry contains data on all recorded deaths of game species and some other wildlife species. For each individual, information on sex, age, location, and estimated cause of death (including the category “disease”) is available. We also used a national digital map of local population density of chamois (for description and methods see Adamič et al. [5] and Flajšman et al. [29]). Considering the purpose of the analysis and limitations of the available data, we used different methods/tests for statistical analysis. The presence of temporal trends in the frequency of chamois submitted for analysis was analyzed with linear regression, temporal trends of changes in proportions were analyzed using non-parametric correlation, and differences in sex and age structure between carcasses submitted for study and all recorded cases of natural mortality were analyzed using tests for homogeneity of structures (Chi-squared statistics). All statistical analyzes were performed using Statistica 10.0 (StatSoft, Inc., Tulsa, OK, USA).

## 3. Results

In the period 2000–2020, a total of 284 chamois carcasses were submitted from chamois hunting areas in Slovenia. We performed 284 necropsies, 153 bacterial identifications, 284 parasitological tests, 198 histopathological tests, and 12 PCR tests for detection of ORFV. The primary causes of death determined by necropsy and various laboratory methods are listed in Table 1 and Table 2.

Death occurred in 28 animals as a result of trauma. These deaths were the result of road traffic accidents (*n* = 14), fall trauma (*n* = 10), firearms (*n* = 3), or predators (*n* = 1). The most common lesions in the trauma category were tissue lacerations and perforations, bone fractures, and severe hemorrhages.

Metabolic disorders (*n* = 2) included ruminal acidosis caused by excessive grain consumption. The major gross lesions in these two cases of ruminal acidosis were swollen and edematous rumen papillae, hyperemia and hemorrhage of the mucous membrane, congestion and edema of the lungs, congestion of the brain and liver, and peritonitis.

Neoplasms of various origins were diagnosed in three cases (cutaneous fibrosarcoma, cutaneous myxofibroma, hepatic cholangiocarcinoma). In one case, winter starvation was another non-infectious cause of the chamois disease.

Parasitic infections, diagnosed in 199 cases (70.3%), were the dominant group within the infectious diseases. Depending on the worm species, parasites were found in the lungs and trachea, as well as in the stomach, small intestine, cecum, and colon contents. Parasitic infections categorized as a cause of death or disease mainly affected the gastrointestinal tract and were associated with enteritis, while in the respiratory tract they were associated with bronchitis or verminous pneumonia. In some cases, secondary bacterial infections occurred. Necropsy in the case of ectoparasites, especially sarcoptic mange, usually resulted in crusts, alopecia, dermal fissures, inflammation of the eyelids and lips, hyperpigmentation and lichenification, and weight loss. An overview of the parasite species identified in chamois is given in Table 2. *Sarcoptes scabiei* was the main cause of mortality (42.6%). The majority (82.3%) of infected chamois in the present study harbored two or more species of helminth parasites. The predominant group of helminths were nematodes. The identified nematode species were *Haemonchus contortus*, *Spiculopteragia asymmetrica*, *Skrjabinagia kolchida*, *Ostertagia leptospicularis*, *O. ostertagi*, and *O. circumcincta*, all in the abomasum; *Trichostrongylus axei*, *T. vitrinus*, *T. colubriformis*, and *Capillaria bovis* in the abomasum and small intestine; *Cooperia* spp. in the small intestine; *Trichuris ovis* in the cecum; *Chabertia ovina* and *Oesophagostomum venulosum* in the large intestine; and *Muellerius capillaris*, *Neostrongylus linearis*, *Protostrongylus* spp., and *Dictyocaulus viviparus*, all in the lungs. Cestodes included *Taenia hydatigena* and *Moniezia expansa*. Ectoparasite fauna included one tick species (*Ixodes ricinus*) and one louse species (*Damalinia meyeri*). During the 21 years of sampling, the predominant intestinal nematode species were *H. contortus* and, in the lungs, *M. capillaris*.

Various bacterial species were responsible for 26 chamois infections (9.3%). The infections were attributed to eight bacterial species or, in one case, to a mixed bacterial flora (see Table 2). The diagnosis of bacterial infection was based on isolation of the bacteria, identification of the pathogens, and corresponding pathologic lesions during histopathologic examination.

During the study period, the total registered annual mortality (whose main cause was harvesting, followed by mange and other diseases) in chamois in Slovenia averaged 2.435 animals (CI 95%: 2.343–2.528). The number of registered chamois that died annually presumably due to disease, based on visual identification of carcasses by hunters, was 80 (CI 95%: 64–96). In addition, an annual average of 99 deaths (CI 95%: 75–122) were reported where hunters were unable to self-assess the cause of death, a significant proportion of which were likely due to disease. Assuming that about one-fifth of the natural mortality of chamois is recorded, as previous studies in Slovenia have shown, the actual annual mortality of chamois is about 400 (approx. 80 × 5), but probably closer to 900 (approx. 5 × (80 + 99)). During the same period, hunters submitted samples of 13.5 (CI 95%: 11–16) animals per year, suggesting that our study covers only about 1.5% of the total disease-related mortality in chamois in Slovenia.

Annually, 4–22 animals were submitted for examination by hunters, and this number did not change systematically (following a linear trend) during the study period (β = −0.33, *p* = 0.11, *n* = 21). The number of samples provided compared to total recorded mortality and total recorded natural mortality of chamois also showed no apparent temporal trend.

**Figure 1:**
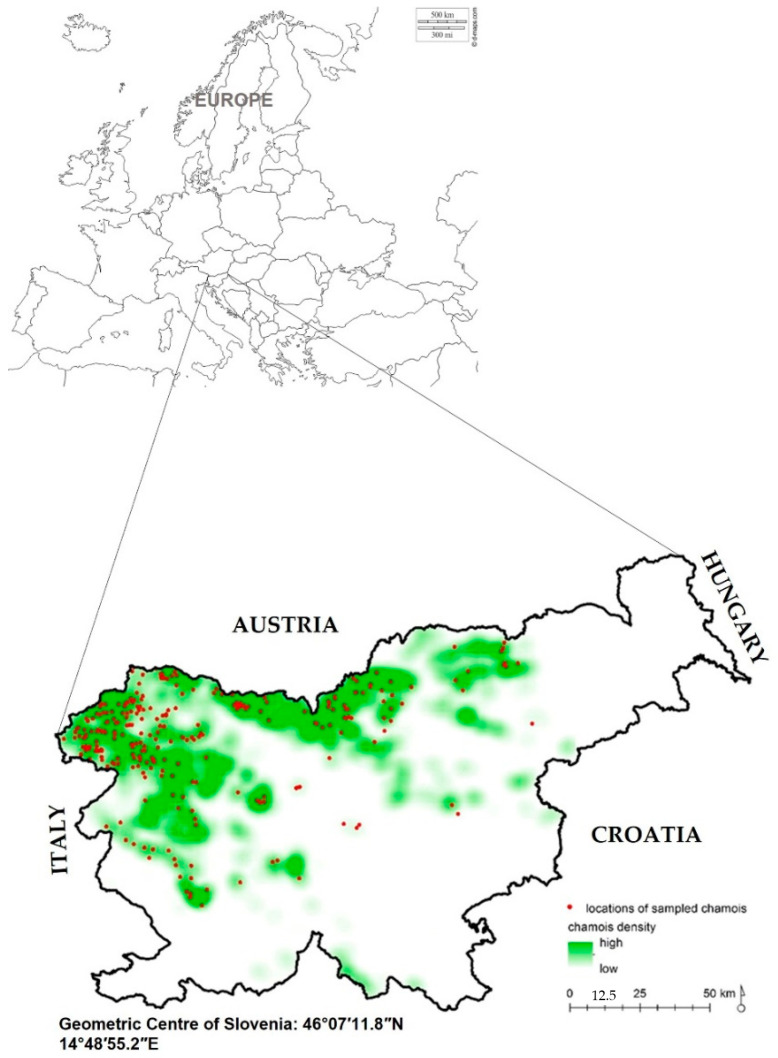
Map of Slovenia with marked locations of sampled and analyzed free-ranging chamois (*n* = 284; red dots) and relative population density of chamois (lowest to highest).

The chamois provided for the study originate from 68 of the total 237 hunting grounds covering the entire home range of the species in Slovenia. The hunting grounds from which the samples were collected host about 77% of the chamois population in Slovenia and an even larger proportion of the total Slovenian territory with permanent presence and reproduction of chamois. Samples were collected mainly in hunting grounds where the density of chamois is relatively high, indicating that the sampling practically covers most of the core habitat of the chamois in the country (see also Figure 1).

The age and sex structure of the supplied samples differed from the structure of all recorded animals whose mortality was recognized as disease by the hunters. Samples supplied had a higher proportion of males (64% vs. 53%), a slightly lower proportion of adults (over 2 years: 60% vs. 57%), and a higher proportion of juveniles (1–2 years; 16% vs. 20%) compared to all the documented natural mortalities. Overall, adult females were highly underrepresented in the samples provided (20% of all laboratory samples versus 30% of all documented cases of natural mortality). Thus, the difference in structure suggests that the samples provided were not random, but the result of a specific selection by hunting ground managers.

## 4. Discussion

To our knowledge, this is the first study on the causes of death of chamois in Europe based on passive monitoring. Passive surveillance of wildlife health aims to detect the presence or spread of disease or infection, or the early detection of emerging or re-emerging diseases in a country, and can provide valuable information for national surveillance systems [30]. A necropsy is required to determine the cause of death or disease in an animal. This examination identifies tissue lesions and paves the way for appropriate additional investigations, such as bacteriological or parasitological examinations [31]. Wildlife health studies based on post-mortem examinations of wildlife carcasses are associated with numerous potential problems related to the detection of diseased or dead animals, quality of material, sample collection, interpretation of results, etc. [32,33,34]. Predation, scavenging, and carcass decomposition can interfere with the observation of dead animals and usually complicate the diagnostic process when carcasses are found [31,34,35]. In our opinion, sampling by hunters was probably not systematic and unbiased. They likely sent for necropsy the cases they thought would be interesting (animals that had unusual symptoms or behavior or were potential trophy carriers) and carcasses that were not yet too decomposed. Nevertheless, passive health surveillance of wildlife is a valuable source of information on the causes of mortality, disease susceptibility, and host pathology and is considered an essential component of early warning systems [31].

Because of the distortion of the samples provided as described above, the relevance of specific diseases to population mortality is given only in general terms (in terms of size classes). Therefore, our monitoring cannot be considered an objective indicator of specific disease-related mortality of chamois in Slovenia. Nevertheless, the number of animals in absolute numbers was high, which is particularly important because, in clinicopathological studies, all disease processes affecting the studied animals are determined [31,36].

During the period 2000–2020, we diagnosed several pathogens causing sporadic infections in chamois. None of the identified diseases was considered a significant threat to animal or human health.

In this study, infectious diseases were identified as the leading cause of death more frequently (82.2%) than non-infectious diseases (11.8%).

Parasitic infections were responsible for 199 (70.3%) of the diseases diagnosed in the chamois in Slovenia. The majority (82.3%) of infected chamois in the present study harbored multiple helminth species. Our data are in agreement with the results of other studies on parasitic species in chamois in Europe [37,38,39,40]. Several studies have shown that parasites can have an impact on the health status of natural ruminant populations [39,41,42]. *Sarcoptes scabiei* was the major cause of mortality in chamois (42.6%) in Slovenia and is probably the most severe disease-affecting wild Caprinae in Europe [4]. In addition, sarcoptic mange can lead to a drastic reduction in, and even prohibition of, any harvest in the affected wildlife management units [4,6]. Mixed infections with different species of *trichostrogylids* and *protostrongylids* were the second most important cause of mortality in chamois, followed by pulmonary parasites. Our data are in agreement with the results reported by Kanchev [43] in Bulgaria in a herd of Balkan chamois. Lungworm infections cause severe and often fatal disease in chamois. *Protostrongylid* nematodes are common in chamois in Slovenia, but the only available work is that of Brglez et al. [44]. In the present study, 9.5% (27/284) of chamois died from verminous pneumonia. As in previous studies in Slovenia, *M. capillaris* and *N. linearis* were the predominant parasites associated with extensive nodular lesions. In addition, the genera *Neostrongylus* and *Muellerius* appear to be well adapted to chamois and are most frequently mentioned by many authors [37,43,45,46,47].

Species of *Pasteurellaceae* have been documented in a number of wild and domestic ungulate species with cases of hemorrhagic septicemia and bronchopneumonia [48]. Pasteurellaceae are part of the normal flora of the upper respiratory tract of healthy animals. However, when the immune response is compromised, such as during periods of stress or when secondary viral or parasitic infections occur, *Pasteurellaceae* species can cause local or systemic disease [49]. According to Wolf [50], infection with *Pasteurellaceae* can cause large losses in wildlife populations. We have demonstrated the presence of *Bibersteinia trehalosi* and *Mannheimia granulomatis* in chamois. The overall prevalence in Slovenia was low (4/284; 1.4%), but a sudden die-off due to *Mannheimia* and *Bibersteinia* strains in northeastern Austria resulted in a decline in the chamois population by about 30% [8]. *Corynebacterium pseudotuberculosis* is the etiological agent of caseous lymphadenitis (CLA) in domestic and wild ruminants. The disease also affects humans, especially those who have had occupational contact with infected animals (e.g., foresters, hunters, or veterinarians) [11,51,52]. The overall prevalence in Slovenia over the last 21 years was 1.4% (4/284). In alpine chamois, CLA was also detected in Italy in two slaughtered animals with paraplegia [53]. Later, 98 cases of CLA were studied in alpine chamois collected over a 16-year period, although they were not necessarily associated with animal death [11].

Concomitant infections were responsible for 9 (3.2%) of the diseases diagnosed in chamois in Slovenia. Concomitant infections are common in animals and often associated with interactions between helminths and bacteria [54]. Co-infections in wildlife can be investigated using classical approaches based on sample collection, detection of infectious agents, and analysis of the results [55]. In necropsy, the different helminths and bacteria can be distinguished, which allows us to identify simultaneous or concomitant infections [56,57].

This study shows that contagious ecthyma occurs occasionally in the chamois population (8/284; 2.8%). Contagious ecthyma is endemic worldwide but is rarely mentioned in the literature because of its low morbidity and minimal economic impact [58]. The disease can become a serious problem in young, stressed, immunocompromised, or overcrowded animals [59,60]. In Europe, sporadic clinical forms of contagious ecthyma have been reported in chamois in Germany [61], Austria [20], and Italy [62], while seropositive results have been reported in Spain [63] and Italy [9]. It is not known whether the Orf virus is endemic in the chamois population or whether the virus is occasionally introduced from local goat or sheep herds. One possibility is an introduction by domestic goats and sheep grazing on the same pastures as the wild animals [20,64]. As in sheep [65], there is also a hypothesis that subclinical parapoxvirus infections can cause outbreaks in wild populations under favorable conditions [66]. Such transmission has been observed in red deer [66] and muskox [64].

Trauma is the main diagnosis of noninfectious origin in chamois. In this study, 9.8% (28/284) of the chamois died due to trauma, of which 50% were identified as traffic accidents, and 35.7% were caused by fall trauma or avalanches as the main diagnosis. The predation rate was marginal (1/284). Predator cases are generally not found when the prey is small and/or the carcass was quickly completely consumed [32]. When a carcass is discovered, hunter motivation to submit it is higher for unusual or unclear deaths than for known causes, such as roadkill or disease characterized by typical macroscopic lesions [21].

Reports of tumors in free-ranging chamois are very rare. Compared to domestic animals, neoplasms are rarely observed in free-living animals, and reports mostly refer to histopathological findings in dead animals [12]. There are only four reports of neoplasms in chamois in the literature, adenomatous neoplasms of the gallbladder [67], a fibroblastic osteosarcoma [68], a nasal papilloma [13], and an astrocytoma [12]. In this study, cutaneous fibrosarcoma, cutaneous myxofibroma, and hepatic cholangiocarcinoma were diagnosed, which have a mortality rate of 1% in the chamois in Slovenia.

## 5. Conclusions

This paper represents the first large-scale study of chamois mortality in Slovenia and provides an overview of the health status of the Slovenian chamois population through passive population monitoring. Surveillance of the health status of wild populations and monitoring for disease outbreaks is important both for the welfare of wild populations and for protecting the health of domestic animals and humans. The results of the present study indicate that although there is a wide range of diseases in the chamois population, none of them threaten the existence of the chamois population in this region. None of the described pathogens pose a major health threat to other wild populations or to humans. Passive disease control is of great importance for clinical and pathological studies that can provide information on disease processes in the species studied, although it cannot on its own be considered an objective indicator of specific disease-related mortality in chamois.

## Figures and Tables

**Table 1:** Primary causes of death in chamois in Slovenia, 2000–2020, determined by necropsy and additional laboratory methods.

Primary Cause of Death	Number	%
Bacterial infections	26	9.3
Viral infections	8	2.8
Metabolic disorder	2	0.7
Neoplasia	3	1
Parasitic infections	199	70.3
Trauma	28	9.7
Winter starvation	1	0.3
Undetermined	17	6
Total	284	100

**Table 2:** Overview of detailed causes of death or emergency removals associated with the primary disease diagnosed in chamois in Slovenia, 2000–2020.

Cause of Death/Emergency Removal	Male	Female	Kids and Juveniles (below 1 Year)	Juveniles (1–2 Years Old)	Adults (over 2 Years)	Total	%
Non-infectious diseases							
Metabolic disorder	-	2	-	-	2	2	0.7
Neoplasia	1	2		-	3	3	1
Trauma	18	10	1	8	19	28	9.8
Winter starvation	-	1	-	-	1	1	0.3
Total	19	15	1	8	25	34	11.8
Infectious diseases							
Bacterial							
*Serratia marcescens*	1	1	-	-	2	2	0.7
*Pseudomonas* *aeruginosa*	1		-	-	1	1	0.3
*Mycoplasma* spp.	1	1	-	-	2	2	0.7
*Bibersteinia trehalosi*	3		-	1	2	3	1
*Staphylococcus* *aureus*	1		-	1	-	1	0.3
*Mannheimia* *granulomatis*	-	1	-	1	-	1	0.3
*Corynebacterium pseudotuberculosis*	3	1	-	2	2	4	1.4
*Yersinia pseudotuberculosis*		1	-	-	1	1	0.3
Mixed bacterial flora		1	-	-	1	1	0.3
Viral							
Contagious ecthyma (PCR)	2	2	2	1	1	4	2.8
Contagious ecthyma (only histological)	2	2	1	1	2	4	
Concomitant infections (bacteria/parasite)	8	1			9	9	3.2
Parasitic							
*Sarcoptes scabiei*	88	33	6	26	89	121	42.6
*Haemonchus* *contortus*	3	1	1	2	1	4	1.4
Protostrongylidae	14	13	3	10	14	27	9.5
*Chabertia ovina*	1	3	-	1	3	4	1.4
Multiple endoparasitism	24	20	5	22	17	44	15.1
Total	152	81	18	68	147	233	82.2
Undetermined cause	11	6		2	15	17	6
Total	182	102	19	78	187	284	100

## Data Availability

The data presented in this study are available upon request from the corresponding author.
